# Large Renal Calculus in a Patient With Crossed Fused Ectopic Kidney Managed With Percutaneous Nephrolithotomy: A Case Report

**DOI:** 10.7759/cureus.73294

**Published:** 2024-11-08

**Authors:** Shashikant Asabe, Ashish Gavade, Vilas P Sabale

**Affiliations:** 1 Urology, Dr. D. Y. Patil Medical College, Hospital and Research Centre, Dr. D. Y. Patil Vidyapeeth (Deemed to be University), Pune, IND

**Keywords:** congenital anomaly, crossed fused renal ectopia, left to right, pcnl, urolithiasis

## Abstract

Congenital anomalies are not regularly seen in day-to-day practice. Crossed fused renal anomalies are even rarer. A 50-year-old female patient presented with right-sided pain in the abdomen with intermittent episodes of burning micturition. The patient was a known case of Mayer-Rokitansky-Kuster-Hauser syndrome. X-ray of the kidney, ureter, and bladder showed right-sided renal calculus. Computed tomography showed non-visualization of the left kidney in the left renal fossa but was crossed over to the right kidney with right renal pelvic calculus. A diagnosis of crossed fused kidney with calculus disease was made. Percutaneous nephrolithotomy was performed. With advancements in minimally invasive surgeries and imaging techniques, such large renal calculus in anomalous kidneys can be managed with good results.

## Introduction

Congenital anomalies are rare in kidneys as most remain asymptomatic. Almost 10-12% of anomalies are identified in adulthood. Congenital renal anomalies might be seen as an absent kidney, increased number of kidneys, malrotation, change in position, shape, fusion, and other urinary tract anomalies [1]. Ectopic kidneys can be at any location, such as thoracic, pelvic, or crossed fused renal ectopia (CFRE) [2].

The incidence of CFRE on autopsy and live birth has been reported to be approximately 1:7,500 and 1:1,000, respectively [3]. Often, these anomalies are associated with a few complications such as hydronephrosis, recurrent urinary tract infections, a high incidence of stone formation, abdominal mass, and other congenital anomalies. A few cases of Wilm’s tumor and renal cell carcinoma along with CFRE have also been reported [4,5]. The male-to-female prevalence ratio of this condition has been reported to be 3:2. Left-to-right ectopia is more common than right-to-left ectopia. The left-to-right fusion ectopia ratio is 3:1 [6].

CFRE remains undiagnosed until symptoms such as abdominal pain, dysuria, and hematuria are reported [7]. On ultrasound, a characteristic notch between two fused kidneys can be seen [8]. Infection, nephrolithiasis, pelvic-ureteric junction obstruction (PUJO), and neoplasia are a few complications of CFRE [9].

## Case presentation

A 50-year-old female patient presented with right-sided pain in the abdomen with intermittent episodes of burning micturition. The pain was intermittent in nature and was relieved with medications. There was no history of dysuria or hematuria, lower urinary tract symptoms, and fever. The patient was a known case of Mayer-Rokitansky-Kuster-Hauser syndrome (MRKH). She was also hypertensive. There was a history of diagnostic laparoscopy for MRKH 30 years back and a history of right ureterorenoscopic lithotripsy 10 years back.

On medical examination, pulse was 76 beats/minute, blood pressure was 106/76 mmHg, temperature was 98.6°F, and saturation was 97% on room air. There was no icterus, clubbing, cyanosis, pallor, or lymphadenopathy. The systemic and local examinations were normal.

Laboratory investigations revealed that serum creatinine was within the normal limits at 0.62 mg/dL. Other tests including complete blood count, liver function test, and blood glucose were within the normal limits.

**Figure 1:**
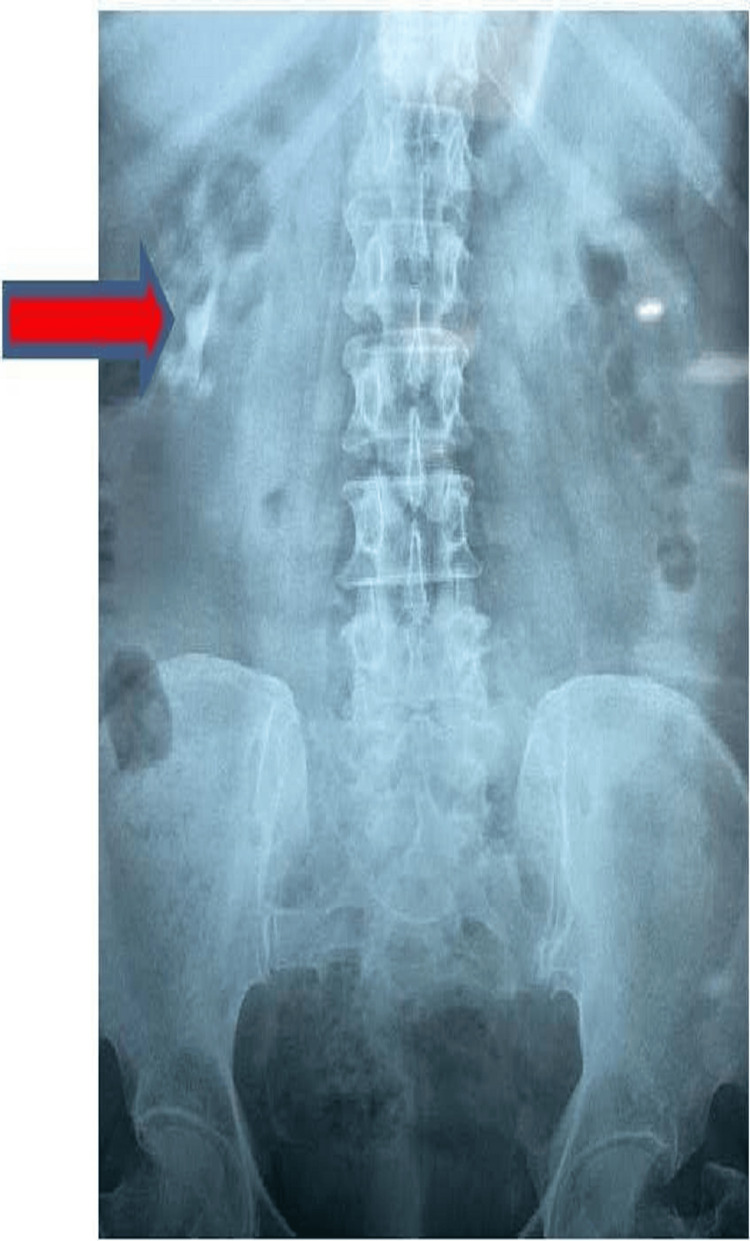
X-ray of the kidney, ureter, and bladder. The arrow shows the right-sided renal calculus.

X-ray of the kidney, ureter, and bladder (KUB) showed right-sided renal calculus (Figure 1). CT showed non-visualization of the left kidney in the left renal fossa but was crossed over to the right side. Both kidneys were malrotated. The left ureter was crossing the midline. Both vesicoureteric junctions were at normal positions.

**Figure 2:**
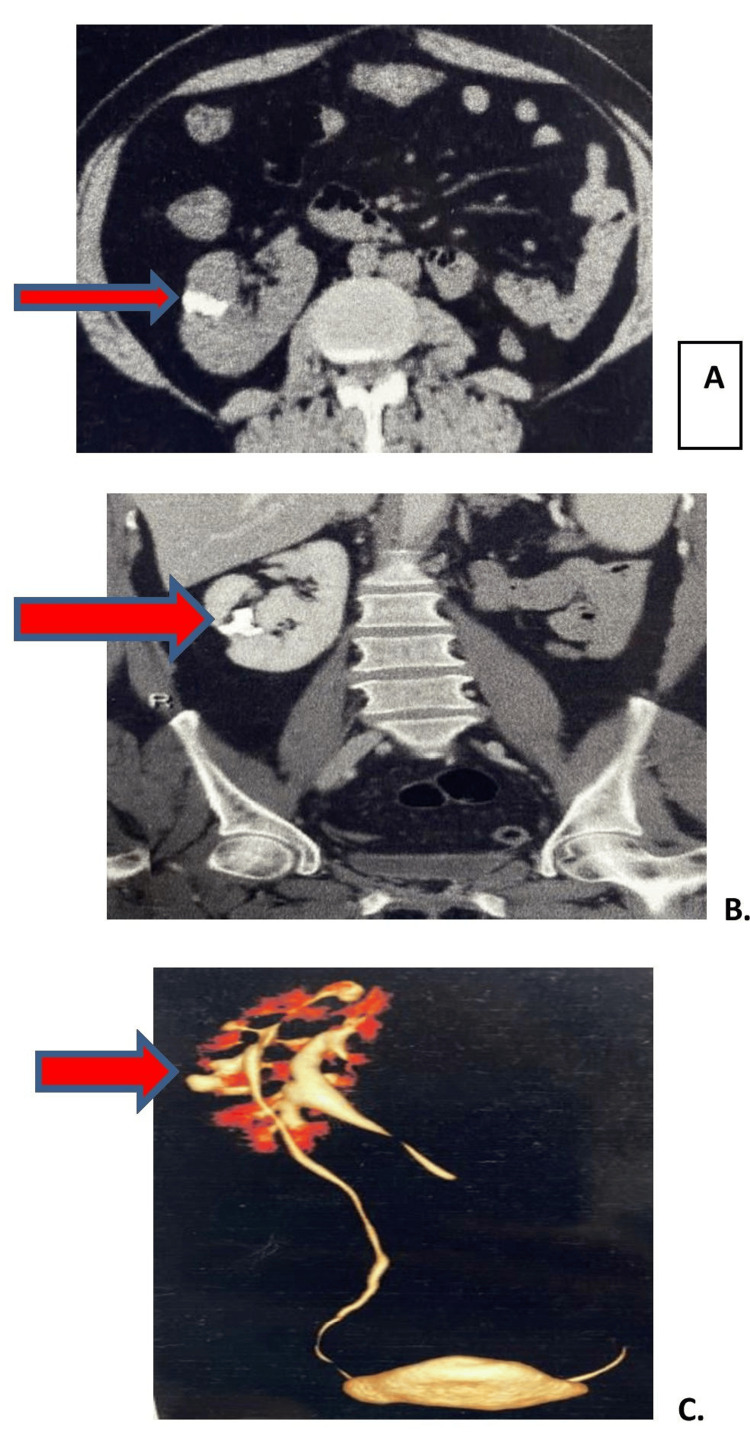
CT images. (A) The arrow in the axial cut of the CT scan shows the right renal calculus. (B) Coronal image of the CT scan shows the right renal calculus. (C) Reconstructive CT image shows the left kidney crossed and fused to the right side, with the left ureter crossing over from the right to the left side which is usual in the case of crossed fused kidneys.

A CT scan showed right renal pelvic calculus measuring 22 × 21 × 27 mm of 900-1,000 Hounsfield unit (HU) (Figure 2). Both kidneys were supplied by a single artery and drained by a single renal vein. There was non-visualization of the uterus, cervix, and the upper two-thirds of the vagina, likely suggestive of Mullerian agenesis. The patient was planned for a minimally invasive procedure, percutaneous nephrolithotomy (PCNL).

**Figure 3:**
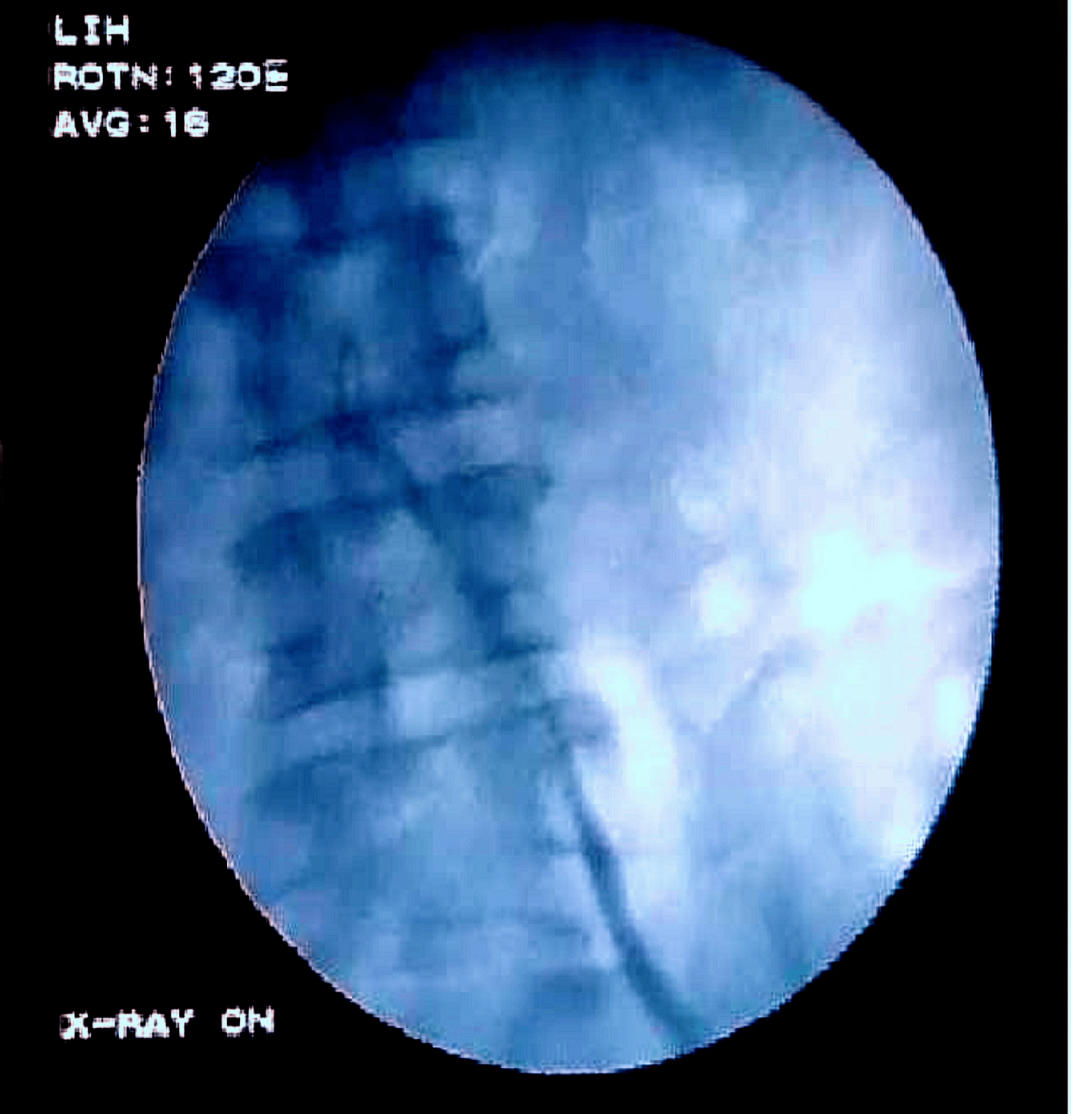
Left retrograde pyelography. Retrograde pyelogram showing the left ureter crossing from the left to the right side.

**Figure 4:**
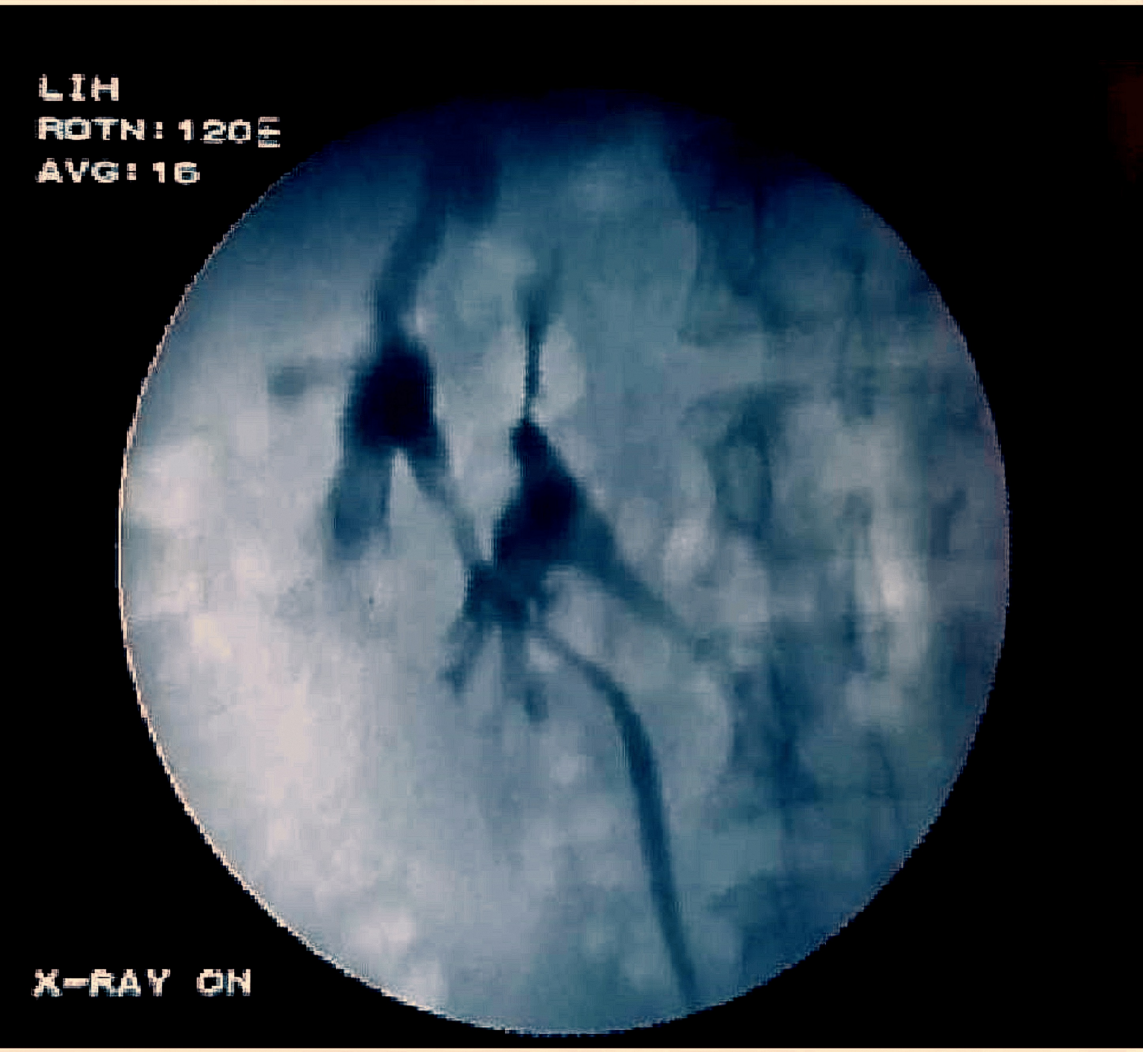
Right retrograde pyelography. Right retrograde pyelogram showing the right kidney adjacent to the left kidney.

Cystoscopy showed bilateral ureteric orifices at normal positions. Left retrograde pyelography showed the ureter crossing over the right side (Figure 3). Right-sided retrograde pyelography was performed (Figure 4) which showed the right kidney adjacent to the left kidney.

PCNL was performed and the following challenges were encountered: (1) abnormal anatomy, (2) possible colon injury, (3) possible blood vessel injury, and (4) possible injury to adjacent organs.

**Figure 5:**
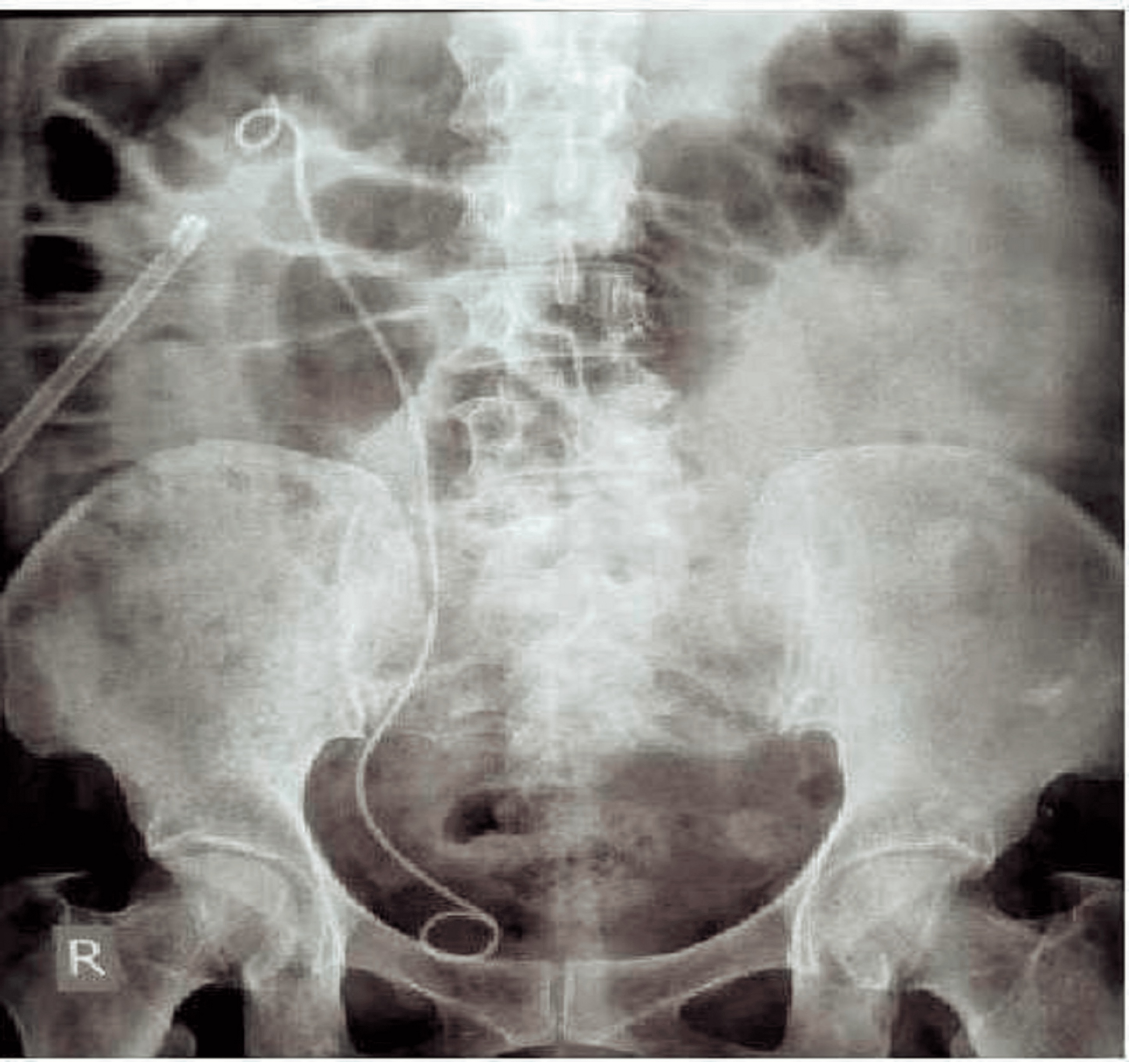
Postoperative X-ray of the kidney, ureter, and bladder suggestive of no residual calculus.

Complete C-arm stone clearance was achieved. A double J stent was placed. Postoperative X-ray KUB showed no residual calculus (Figure 5).

## Discussion

After horseshoe kidney, CFRE is the second most common fusion anomaly [10]. Open surgery is frequently used to treat anomalous kidneys in these patients given abnormal anatomy and better visualization of anatomical structures. Nonetheless, a laparoscopic method for treating this anomaly has been documented [11-14]. For a variety of purposes, transperitoneal and retroperitoneoscopic [13] techniques have been employed. Transperitoneal access offers greater room, improved visibility, and better handling of vasculature, which is why the majority of authors have employed it. On the contrary, retroperitoneal access has no bowel handling.

Because of the aberrant position, malrotation, and relationship between the kidney and the intestine and vertebral column, managing renal stones in patients with CFRE can be challenging. Conventional therapy options, such as PCNL and extracorporeal shockwave lithotripsy (ESWL), may not always be appropriate or effective, though they have been reported. Although ESWL has been described, the presence of underlying bone and overlying intestinal gases may make it technically challenging [15,16].

Because of malrotation or associated PUJO, stone clearance following fragmentation may also be less than optimal. Three of the 150 reported cases of anomalous kidneys with renal stones reported by Tunc et al. had CFRE [16]. In these circumstances, the outcome was a mere 25%. Table 1 summarizes similar cases reported in the literature.

**Table 1:** Similar cases reported in the literature.

Author	Age (years)/Sex	Radiological findings	Treatment
Cao et al. [3]	59/F and 24/F	Case 1: Right-to-left crossed fused ectopic kidney and calculi seen in the left kidney. Case 2: Right kidney crossed to left with two 3-mm calculi in both kidneys	Percutaneous nephrolithotomy was performed for the first case. Conservative management was adopted for the second case.
Aminsharifi et al. [12]	32/M	Left-to-right crossed and fused right kidney and calculus seen in the left kidney	Laparoscopic nephrolithotomy was performed
Resorlu et al. [17]	28/M	Right-to-left crossed fused kidney	Retrograde intrarenal surgery was performed

## Conclusions

We presented the case of a 50-year-old female with a crossed fused ectopic kidney with renal calculus and MRKH. Such a case is rare in day-to-day practice. Advanced imaging modalities help in diagnosing such complicated cases and can guide management. Using newer endoscopic instruments, such as smaller caliber nephroscopes with improved vision, such large calculus can be managed with smaller incisions. Hence, open surgical scars can be avoided with associated perioperative pain and delayed postoperative recovery.
